# Combined Effect of Early Nutrition Therapy and Rehabilitation for Patients with Chronic Obstructive Pulmonary Disease Exacerbation: A Prospective Randomized Controlled Trial

**DOI:** 10.3390/nu16050739

**Published:** 2024-03-05

**Authors:** Yohei Oyama, Hiroomi Tatsumi, Hiroko Takikawa, Natsuko Taniguchi, Yoshiki Masuda

**Affiliations:** 1 Department of Intensive Care Medicine, Sapporo Medical University School of Medicine, Sapporo 060-8543, Japan; htatsumi@sapmed.ac.jp (H.T.); ymasuda@sapmed.ac.jp (Y.M.); 2 Division of Rehabilitation, Japan Community Health care Organization Hokkaido Hospital, Sapporo 062-8618, Japan; 3 Division of Nutrition Management, Japan Community Health Care Organization Hokkaido Hospital, Sapporo 062-8618, Japan; pyun470817@gmail.com; 4 Respiratory Disease Center (Pulmonary Medicine), Japan Community Health Care Organization Hokkaido Hospital, Sapporo 062-8618, Japan; taniguchi-natsuko@hokkaido.jcho.go.jp

**Keywords:** chronic obstructive pulmonary disease, exacerbation, nutrition therapy, rehabilitation program

## Abstract

The effectiveness of rehabilitation programs (RP) for chronic obstructive pulmonary disease (COPD) exacerbation remains controversial. However, few studies have investigated the combined effects of exercise and nutritional therapy. This study aimed to determine the effects of combined nutritional therapy on the physical function and nutritional status of patients with COPD exacerbation who underwent early RP. A randomized controlled trial was conducted in patients hospitalized for COPD exacerbations. Patients were assigned to receive a regular diet in addition to RP (control group) or RP and nutrition therapy (intervention group). Physical function, including quadricep strength and body composition, was assessed. The intervention group was administered protein-rich oral nutritional supplements. A total of 38 patients with negligible baseline differences were included in the analysis. The intervention group showed a notably greater change in quadriceps strength. Lean body mass and skeletal muscle indices markedly decreased in the control group but were maintained in the intervention group. Logistic regression analysis identified nutritional therapy as a significant factor associated with increased muscle strength. No serious adverse events were observed in either group. Therefore, nutritional therapy combined with RP is safe and effective for improving exercise function while maintaining body composition in patients with COPD exacerbation.

## 1. Introduction

Chronic obstructive pulmonary disease (COPD) is a common condition characterized by persistent respiratory symptoms and airflow limitation [1]. It develops following long-term exposure to harmful substances, such as cigarette smoke, which causes inflammation of the airways and alveoli. Moreover, it is associated with systemic symptoms such as malnutrition and skeletal muscle dysfunction. The prevalence and mortality rates of COPD are high worldwide. COPD was ranked as the third leading cause of death in a World Health Organization survey [2].

In stable COPD, rehabilitation programs (RPs) are recommended in addition to drug therapy with long-acting inhaled bronchodilators. Several high-quality studies have investigated the effects of rehabilitation on COPD, revealing outcomes such as reduced number of exacerbations and length of hospital stay, increased skeletal muscle strength, as well as improved exercise tolerance and quality of life (QOL) [3,4,5,6]. Stable COPD patients are often malnourished. Malnutrition is associated with emphysematous changes and airflow limitation [7,8,9]. Recently, the effects of nutritional interventions on undernutrition in patients with COPD have been a focus of concern, and weight gain, lean body mass gain, improvements in QOL, exercise tolerance, and respiratory and muscle strength have been reported [3]. Furthermore, weight loss is an independent prognostic factor in patients with COPD [10,11,12,13].

Conversely, exacerbations are common in patients with COPD. Short-acting inhaled bronchodilators, antibiotics, and corticosteroids are used to treat patients with exacerbated COPD. Additionally, oxygen and non-invasive mechanical ventilation therapies are used as non-pharmacotherapies. There have been positive reports on the effects of RP as non-pharmacological therapy for COPD exacerbation, including improved exercise tolerance, muscle strength, and QOL, as well as shorter hospital stays [14,15,16,17]. However, these findings are controversial and inconclusive. Furthermore, few studies have investigated the combined effects of exercise and nutritional therapy in patients with COPD exacerbations.

This study aimed to determine the effects of nutritional therapy on the physical function and nutritional status of patients with COPD exacerbation who underwent early RP. 

## 2. Materials and Methods

### 2.1. Statement of Ethics

This study was conducted in compliance with the ethical guidelines for medical research involving human participants and in accordance with the principles set forth in the Declaration of Helsinki. The participants were provided a full explanation in advance by the researcher, and those who agreed to participate provided written informed consent. Participants who did not provide informed consent were excluded from the study. This study was approved by the Ethics Review Committee of the Japan Regional Medical Organization Hokkaido Hospital (approval number 2019-11, 19 August 2019) and registered in the public clinical trial registry (registration number of clinical trial, UMIN000037984) before the first patient was included. The study protocol describes the detailed course of the study and is available online on the website “https://center6.umin.ac.jp/cgi-open-bin/ctr/ctr_view.cgi?recptno=R000043285 (accessed on 30 October 2023)”.

### 2.2. Study Participants

Patients with COPD exacerbation who were admitted to JCHO Hokkaido Hospital between 1 September 2019 and 31 August 2023, and who provided consent for our study, were included. The exclusion criteria were as follows: ventilator support for >48 h, difficulty with oral intake due to dysphagia or other problems, inability to walk due to musculoskeletal or cerebrovascular disease, cancer without curative treatment, interstitial pneumonia, continuous steroid medication before admission, severe visual or hearing impairment, and dementia or delirium.

During the study period, patients who declined to participate in the study, had serious adverse events, had difficulty with oral intake during part or the entirety of the study period, had a short hospital stay, or were discharged within seven days of the start of RP were also excluded.

### 2.3. Study Design

This was a prospective, open-label, randomized controlled trial. Participants were assigned in a 1:1 ratio to a control group that received early rehabilitation and the usual diet only, and an intervention group that received early nutrition therapy at the same time as the start of early rehabilitation. Furthermore, stratified random allocation was performed using age, sex, pneumonia status, and long-term oxygen therapy (LTOT) as stratification factors. In this randomized controlled trial, a random number table was implemented to determine the allocation order, and patients were enrolled and assigned by physical therapists who were not involved in data collection or analysis.

### 2.4. Nutrition Therapy

The intervention group received nutritional therapy based on the suggestions of a nutritional support team (NST). The amount of energy provided was the sum of the total energy expenditure (TEE) and the amount of energy expended during exercise in the RP. TEE was calculated by multiplying the basal energy expenditure (BEE), estimated using the Harris–Benedict formula by the activity and stress factors. If the energy intake was less than the BEE, the target energy intake was set as the BEE. Nutritional therapy targeted TEE when the energy intake was equivalent to that of BEE. The activity factor used to calculate TEE was 1.3 at the beginning of exercise therapy and 1.4 when resistance and endurance training were completed. The NST determined a stress factor of 1.1 and 1.3. To efficiently obtain energy, we used HEPAS, an oral nutritional supplement (ONS) (Clinico, Inc., Tokyo, Japan). The ONS used in our study was 125 mL per pack and contained 200 kcal of energy, 6.5 g of protein, and 3500 mg of branched-chain amino acids (BCAAs).

The control group received standard meals, and TEE was calculated based on the Harris–Benedict equation with an activity factor of 1.2 and a stress factor of 1.1.

Supplemental meals consumed by the participants during the study period were reported to the dietitian via a supplemental meal report.

### 2.5. Rehabilitation Programs

RPs were introduced in both groups and generally started within 72 h after admission. However, in some patients, RPs were delayed beyond 72 h after fever, and the inflammatory response peaked. When symptoms, such as dyspnea at rest, were severe and RPs were difficult to perform, RPs were started as soon as symptoms permitted. RPs consisted of patient education and exercise therapy. Exercise therapy included conditioning, resistance, and endurance training. Resistance training consisted of knee extension exercises at 50% 1RM load (three sets of 10 repetitions on each side for a total of 30 repetitions), calf raises, and repetitive chair-standing exercises. Endurance training consisted of 15 min of continuous walking at a speed of 3–5 on the Borg scale. Patients with difficulty sitting and standing were provided with the necessary functional training.

### 2.6. Primary and Secondary Outcomes

The primary outcome of the trial was the change in the quadriceps muscle strength-to-weight ratio. The secondary outcome was changes in skeletal muscle index (SMI).

### 2.7. Data Collection

#### 2.7.1. Basic Characteristics

Data on age, sex, modified Medical Research Council dyspnea score (mMRC score), SpO_2_/FIO_2_ ratio (S/F), LTOT use, complication score (Charlson comorbidity index), presence of infection, steroid use, number of RP sessions during the study period, and length of hospital stay were also extracted. Baseline assessments were performed at the start of the intervention, and the final assessment was performed 15 days later. If discharge occurred earlier than 15 days after the start of intervention, a final assessment was conducted on the day of discharge. 

#### 2.7.2. Physical Function

Quadriceps strength, exercise tolerance using a 6 min walk test and short physical performance battery (SPPB), and walking speed using a 10 m walk test. 

#### 2.7.3. Nutrition Status

Body composition data, including body weight, body mass index (BMI), lean body mass index (LBMI), SMI, and blood test results, were extracted. Energy and protein intakes during the study period were also investigated.

### 2.8. Measurement

#### 2.8.1. Measurement of Physical Function

Quadriceps strength was calculated by dividing the maximum value obtained from two measurements on each side by body weight. A handheld dynamometer (#Tas F1, Anima Inc., Tokyo, Japan) was used for all measurements. Grip strength was measured using a Smedley-type digital grip-strength meter (Grip-D T.K.K. 5101; Takei Kiki Kogyo Inc., Tokyo, Japan); measurements were performed twice in the standing position with both feet shoulder-wide apart. 

#### 2.8.2. Measurement of Nutritional Status

Body composition was measured by bioelectrical impedance analysis using InBody S10 (InBody Japan Inc., Tokyo, Japan). Dietary intake was recorded by a nurse, and a dietitian calculated the energy and protein intakes based on the records. 

### 2.9. Statistical Analysis

We retrospectively surveyed patients with COPD exacerbation at our hospital to determine an appropriate sample size. Data from 68 patients were analyzed. The difference in weight-corrected changes in quadriceps strength between well-nourished and non-nourished patients was 5.8 ± 7.0%. Based on these results, an effect size of 5.8 and a standard deviation of 7.0 were used. The significance level was set at 0.05, and the effect size was set at 0.80. The required sample size was calculated to be 48 cases, and the target sample size was set at 60 cases (30 cases per group) to account for dropouts. 

The Student’s *t*-test, Mann–Whitney U test, and χ^2^ test were performed to test for differences between the two groups. Paired *t*-tests and Wilcoxon signed-rank tests were used to compare the initial and final evaluations of each group. Univariate and multivariate logistic regression analyses were performed to identify the factors influencing changes in muscle strength. For all analyses, statistical significance was set at *p* < 0.05, and JMP Pro version 16.1 (SAS Institute Japan, Tokyo, Japan) was used.

The study period was planned for the last two years, from 1 September 2019 to 31 August 2021 but was extended for another two years due to an insufficient number of enrolled patients. However, despite the extended study period, the planned number of patients was not achieved. The authors received approval to terminate the trial after review by an independent data management committee.

## 3. Results

**Figure 1:**
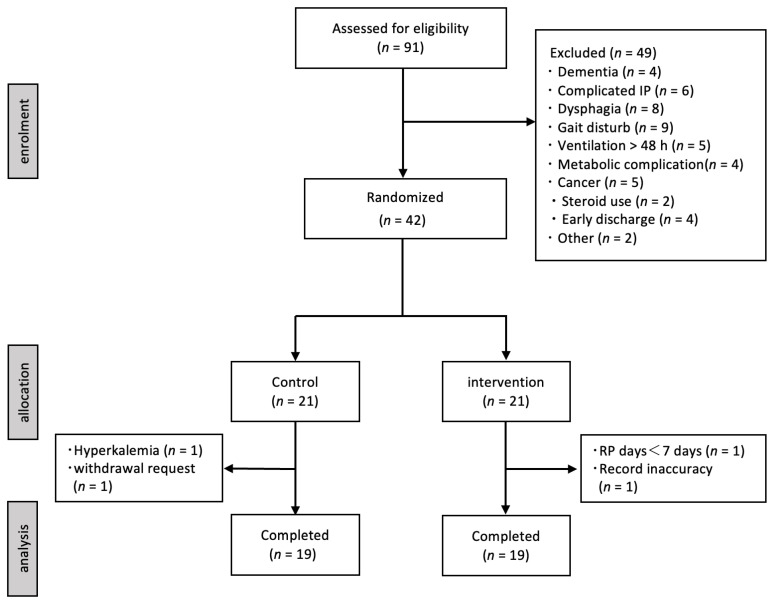
Flow chart of this study. IP; interstitial pneumonia, RP; rehabilitation program.

Ninety-one patients with COPD exacerbations were assessed based on the study inclusion and exclusion criteria. Of these, 49 were excluded, and 42 were included in the study. Two patients in the control group dropped out: one developed hyperkalemia during the study, and the other withdrew from the study. Two patients in the intervention group dropped out of the study. One patient was excluded because he had participated in the study for less than seven days, and one could not be evaluated because of incomplete supplementary feeding reports. Overall, 38 patients were included in the final analysis (Figure 1).

### 3.1. Patient Characteristics

The median age of the patients was 74 [interquartile range (IQR) 68–81] years, and the mean BMI was 20.0 ± 4.1 kg/m^2^ at the start of the study. Table 1 shows the baseline patient characteristics. There were no significant differences between the two groups in terms of age, sex, GOLD category, mMRC score, S/F ratio, LTOT use, presence of infection, or blood test results upon admission. Moreover, there were no differences in the intervention duration, number of sessions, length of stay, baseline nutritional status, or baseline physical function between the two groups. No group changes were observed after the allocation.

### 3.2. Comparison between the Two Groups

The intervention group showed a significantly greater improvement in quadriceps strength, the primary outcome, at 6.9 ± 7.7 kgf/kg compared to 1.4 ± 7.5 kgf/kg in the control group. The intervention group demonstrated significantly greater improvement in gait speed and SPPB scores compared to the control group. There were no significant differences in the changes in the 6 min walking distance.

The intervention group had significantly higher energy and protein intakes during the study period (Table 2). In the control group, there were significant reductions in body weight, LMBI, and the secondary outcome, SMI (−0.3 ± 0.2 vs. 0.1 ± 0.2 kg/m^2^).

### 3.3. Comparison within Each Group

In terms of physical function, quadriceps muscle strength and walking speed improved significantly only in the intervention group (Table 3). The 6 min walking distance and SPPB scores improved significantly in both groups, with significant reductions in body weight, BMI, lean body mass, skeletal muscle mass, SMI, and LBMI in the control group but not in the intervention group.

### 3.4. Univariate and Multivariate Logistic Regression Analyses 

In the regression analysis for predictors of muscle strength improvement, an improvement of 5.2 kgf or more was used as the dependent variable, based on a previous study by Oliveira et al. [18]. The results showed that nutrition therapy was an independent predictor in univariate and multivariate logistic regression analyses (Table 4).

### 3.5. Adverse Event

No serious adverse events related to the early nutritional therapy, such as death or ventilator use, were observed. One patient in the intervention group experienced diarrhea; however, no specific treatment was required. One patient in the control group had hyperkalemia; however, no association with the underlying COPD was noted. There were no significant changes in blood glucose or triglyceride levels in either group and no elevated blood glucose levels requiring treatment (e.g., blood glucose > 250 mg/dL).

## 4. Discussion

Several studies have reported short-term effects of early RPs on COPD exacerbation and physical function. However, patients in the early phase of COPD exacerbation are susceptible to malnutrition owing to an imbalance in energy delivery.

We hypothesized that early nutritional therapy combined with RPs would result in greater functional improvement in patients with COPD exacerbation. The resulting improvement in muscle strength was significantly greater in the nutrition therapy group. In addition, body composition parameters, such as BMI and SMI, decreased in the control group, whereas body composition was maintained in the intervention group. To the best of our knowledge, our study is one of the few on the combined effects of early nutritional therapy and early RPs in patients with COPD exacerbations, and it is the first prospective report of a distinct effect of combined RPs and nutritional therapy on muscle strength in patients with COPD exacerbations.

Zhang et al. conducted a meta-analysis of 14 randomized controlled trials on early RPs in patients with COPD exacerbations [19]. Most patients were in their 60s, and only three studies included participants older than 70 years in both groups; therefore, the participants in our study were considered relatively old. The mean BMI of the participants in this study was 20.0 kg/m^2^, indicating that the study included many patients with nutritional disorders. As the proportion of underweight patients increases in severe COPD, and underweight is an independent prognostic factor [20,21], the results of our study may be an important decision-making aid in the selection of treatments for severe COPD. 

The number of patients in our study did not reach the originally planned 60 because of a decrease in the number of patients with COPD exacerbations owing to the COVID-19 pandemic [22].

The study showed that the intervention was safe, with no serious adverse events; however, the exclusion of patients with severe renal dysfunction or poorly controlled diabetes may have contributed to this result. Additionally, although sarcopenic obesity is prevalent in COPD patients [23], COPD patients in Japan have been reported to have a low BMI [24]. In this study, only one patient had a BMI of 25 or higher. Careful patient selection is important for the clinical implementation of the results of our study.

### 4.1. Nutrition Status

A comparison of nutritional intake between the groups showed significantly higher energy and protein intake in the intervention group. Vermeeren et al. reported that nutritional therapy significantly increased energy and protein intake in hospitalized patients with COPD exacerbation [25]. The same was observed in our study; NST intervention with ONS resulted in higher energy and protein intakes. 

Body weight, LBMI, and SMI were maintained in the intervention group but significantly decreased in the control group. Simone et al. reported that energy and protein intakes improved the body composition of patients with stable COPD [26]. In this study, the same positive effect on body composition was observed in patients with COPD exacerbations. Ogasawara et al. reported that the addition of ONS containing eicosapentaenoic acid to hospitalized patients with COPD exacerbations did not differ from the control group in terms of energy intake but increased protein intake [27]. They reported a trend toward increased SMI in the intervention group, although the difference was not statistically significant. We believe that the higher energy and protein intakes in this study, compared to that in their study, prevented a decrease in body composition, such as SMI. This could be attributed to the fact that the ONS content used in the present study was high among BCAAs.

Within-group comparisons revealed significant reductions in body weight, BMI, lean body mass, skeletal muscle mass, SMI, and LBMI in the control group; however, no significant differences were observed in the intervention group. Previous studies have reported that patients with COPD exacerbation experience a decrease in dietary intake immediately after exacerbation and an increase in resting energy expenditure, resulting in an energy imbalance and a negative balance in energy delivery during the early phase of hospitalization [28]. In this study, we considered effective nutritional therapy with ONS from the early phase of the disease, which corrected the energy imbalance that occurred early in hospitalization and resulted in the maintenance of weight, lean body mass, and skeletal muscle mass, as well as preventing a decrease in SMI and LBMI.

### 4.2. Physical Function

The primary outcome, the change in quadriceps strength, showed significantly greater improvement in the intervention group, confirming our hypothesis. In a systematic review of RPs with ONS by Abdulelah et al., only a few reports on ONS contributed to improvements in lower-extremity muscle strength [26]. However, regarding the relationship between nutritional status and improved physical function, previous studies have reported that protein intake may be more effective in patients with COPD and protein deficiency [29]. In contrast, Shirai et al. reported that approximately half of the Japanese patients with COPD exacerbations had a low BMI and were undernourished [30]. Furthermore, the participants in this study had a low BMI of 20.0 kg/m^2^ and included more malnourished patients, which may have contributed to the effectiveness of nutrition therapy. Therefore, nutritional therapy may be effective in improving muscle strength in patients with COPD exacerbation. 

The results of this study showed that both groups exhibited significant improvements in the 6 min walking distance after RP, and there was no significant difference in the amount of change between the two groups. These results are similar to those reported by He et al. and other studies [31,32], suggesting that early RPs are effective in patients with COPD exacerbations.

SPPB showed significant improvement in both groups in the within-group comparison. However, a significantly greater change was observed in the intervention group in between-group comparisons. Gait speed improved significantly in the intervention group compared to the control group, where no significant improvement was observed. Consequently, the difference in gait speed improvement between the intervention group and the control group was statistically significant. SPPB and gait speed are affected by muscle strength [33,34], and we believe that the increase in muscle strength in the intervention group contributed to these improvements. In our study, we found that a combination of nutritional therapy and RPs positively affected multiple physical functions by improving muscle strength. We also consider that improvements in SPPB performance and gait speed are important results because the deterioration of these parameters is associated with unfavorable outcomes, such as the risk of functional disability, rehospitalization, and death [34,35,36].

### 4.3. Limitations

This study had some limitations. First, the expected number of participants was not met due to the COVID-19 pandemic. Second, the use of a placebo in nutrition therapy could not be blinded. Third, only one type of ONS was used; therefore, the effects of other ONS types could not be determined. Fourth, patients with severe renal dysfunction or poorly controlled diabetes mellitus were excluded from the study; therefore, the effects on these patients could not be confirmed. Additionally, caution should be exercised when generalizing these results to patients with sarcopenic obesity. Finally, long-term events such as death or rehospitalization after discharge were not examined.

## 5. Conclusions

In our study, the combination of early RP- and NST-guided nutritional therapy in patients with COPD exacerbation resulted in greater improvements in quadriceps muscle strength and the maintenance of body composition, including muscle mass, than RPs alone. This suggests that nutritional therapy with ONS, which is high in proteins, particularly BCAA-rich proteins, is a safe and effective intervention for patients with COPD exacerbations. Future large-scale studies should be conducted to identify more effective nutritional therapies by comparing them with other nutritional products. 

## Figures and Tables

**Table 1:** Baseline characteristics of the control and intervention groups.

	Control Group (*n* = 19)	Intervention Group (*n* = 19)	*p*-Value
Age, years (IQR)	74 (70–81)	72 (67–80)	0.518
Sex (male), *n* (%)	14 (73.7)	16 (84.2)	0.693
GOLD category, *n* (A/B/E)	2/12/5	4/9/6	0.677
mMRC score	2 (2–3)	2 (2–3)	0.529
SpO_2_/FIO_2_ ratio	382.7 ± 68.3	395 ± 56.0	0.539
LTOT use, *n* (%)	7 (36.8)	6 (31.6)	1.000
Infection, *n* (%)	11 (57.9)	11 (57.9)	1.000
Charlson index	1.4 ± 0.8	1.4 ± 0.8	1.000
Corticosteroid use, *n* (%)	11 (57.9)	10 (52.6)	1.000
Length of stay, days	17.5 ± 4.9	23.3 ± 20.5	0.236
TP, g/dL	6.3 ± 0.4	6.5 ± 0.5	0.117
Alb, g/dL	3.5 ± 0.8	3.3 ± 0.5	0.434
CRP, mg/dL	3.9 ± 4.3	3.2 ± 4.0	0.603
BW, kg	53.3 ± 14.1	51.8 ± 12.1	0.722
BMI, kg/m^2^ *	20.3 ± 4.0	19.9 ± 4.2	0.754
%QS, kgf/kg	48.0 ± 14.6	48.7 ± 16.0	0.898
6MWD, m	225.1 ± 154.3	255.0 ± 157.9	0.563
10 m timed gait test, sec	9.8 ± 3.6	12.1 ± 4.4	0.095
SPPB, point	9.3 ± 3.2	9.3 ± 3.3	1.000
LMBI, kg/m^2^	14.6 ± 2.0	14.8 ± 2.1	0.745
SMI, kg/m^2^	5.9 ± 2.0	5.8 ± 2.1	0.851

Data are shown as median [25–75% IQR] or mean ± standard deviation. GOLD: Global Initiative for Chronic Obstructive Lung Disease, mMRC score: modified Medical Research Council dyspnea score, SpO_2_: saturation of percutaneous oxygen, FIO_2_: fraction of inspiratory oxygen, LTOT: long-term oxygen therapy, TP: serum total protein, Alb: serum albumin, %QS: percent quadriceps strength, SPPB: short physical performance battery, LMBI: lean body mass index, SMI: skeletal muscle mass index. * Only one participant was obese (BMI ≥ 25).

**Table 2:** Nutritional intake and changes in physical function and body compositions during the study period.

	Control Group (*n* = 19)	Intervention Group (*n* = 19)	*p*-Value
Average nutritional intake			
Energy intake, kcal/kg	28.3 ± 8.4	37.0 ± 11.1	0.015
Protein intake, g/kg	1.2 ± 0.4	1.6 ± 0.4	0.011
Changes in physical function and body compositions			
%QS, kgf/kg	1.4 ± 7.5	6.9 ± 7.7	0.031
6MWD, m	65.8 ± 70.5	57.7 ± 61.0	0.711
10 m timed gait test, sec	−0.5 ± 1.5	−2.5 ± 2.3	0.025
SPPB, point	0.4 ± 1.5	1.4 ± 1.6	0.010
LMBI, kg/m^2^	−0.5 ± 0.5	0.1 ± 0.3	<0.001
SMI, kg/m^2^	−0.3 ± 0.2	0.1 ± 0.2	<0.001
BW, kg	−1.5 ± 1.3	0.3 ± 0.3	<0.001

Data are shown as mean ± standard deviation. TP, serum total protein; Alb, serum albumin; CRP, C-reactive protein; BW, body weight; BMI, body mass index; LMBI, lean body mass index; SMI, skeletal muscle mass index; %QS, percent quadriceps strength; 6 MWD, 6 min walk distance; SPPB, short physical performance battery.

**Table 3:** Nutritional and inflammatory status and physical function at the study baseline and final evaluation.

	Control Group (*n* = 19)			Intervention Group (*n* = 19)		
	Pre	Post	*p*-Value	Pre	Post	*p*-Value
TP, g/dL	6.3 ± 0.4	6.4 ± 0.4	0.183	6.5 ± 0.5	6.6 ± 0.6	0.697
Alb, g/dL	3.5 ± 0.8	3.4 ± 0.5	0.522	3.3 ± 0.5	3.4 ± 0.5	0.576
CRP, mg/dL	3.9 ± 4.3	1.5 ± 2.9	0.025	3.2 ± 4.3	1.3 ± 2.9	0.133
BW, kg	53.3 ± 14.8	51.9 ± 13.6	0.001	51.8 ± 12.1	51.7 ± 11.6	0.915
BMI, kg/m^2^	20.3 ± 4.0	19.7 ± 3.7	<0.001	19.9 ± 4.2	19.8 ± 4.0	0.762
Energy intake, kcal/kg	28.3 ± 8.4	30.5 ± 9.9	0.079	37.0 ± 11.1	38.7 ± 10.1	0.013
Protein intake, g/kg	1.2 ± 0.4	1.3 ± 0.4	0.166	1.6 ± 0.4	1.6 ± 0.4	0.065
LMBI, kg/m^2^	14.6 ± 2.0	14.1 ± 1.9	0.001	14.8 ± 2.1	14.9 ± 2.2	0.252
SMI, kg/m^2^	5.9 ± 1.3	5.6 ± 1.2	<0.001	5.8 ± 1.1	5.9 ± 1.1	0.243
%QS, kgf/kg	48.0 ± 14.6	49.4 ± 16.1	0.438	48.7 ± 16.0	55.6 ± 18.4	0.001
6MWD, m	225 ± 154	291 ± 184	<0.001	241 ± 164	313 ± 176	<0.001
10 m timed gait test, sec	9.8 ± 3.6	9.3 ± 3.8	0.178	12.1 ± 4.4	9.5 ± 2.5	<0.001
SPPB, point	9.3 ± 3.2	9.8 ± 3.3	0.037	9.3 ± 3.3	10.7 ± 3.2	0.001

Data are shown as mean ± standard deviation. TP, serum total protein; Alb, serum albumin; CRP, C-reactive protein; BW, body weight; BMI, body mass index; LMBI, lean body mass index; SMI, skeletal muscle mass index; %QS, percent quadriceps strength; 6 MWD, 6 min walk distance; SPPB, short physical performance battery.

**Table 4:** Univariate and multivariate logistic regression analyses.

	Univariate Regression Analysis			Multivariate Regression Analysis		
	Odds Ratio	95% CI	*p*-Value	Odds Ratio	95% CI	*p*-Value
Age, years	1.025	0.937–1.122	0.584	−	−	−
GOLD category (A,B = 0, E = 1)	1.143	0.264–4.951	0.853	−	−	−
LTOT use, n (yes = 1, no = 0)	0.638	0.137–2.973	0.561	−	−	−
Total steroid use, mg	0.998	0.993–1.002	0.208	−	−	−
CRP, mg/dL	0.859	0.681–1.165	0.145	−	−	−
Number of RP sessions	1.014	0.926–1.110	0.766	1.040	0.135–96.371	0.444
Nutrition therapy (yes = 1, no = 0)	7.65	1.370–−42.713	0.010	7.494	1.336–42.039	0.022

GOLD categories were entered, with 0 for A and B and 1 for E. For LTOT use, users were entered as 1 and non-users as 0. 95% CI, 95% confidence interval; GOLD, Global Initiative for Chronic Obstructive Lung Disease; LTOT, long-term oxygen therapy; CRP, C-reactive protein; RP, rehabilitation program.

## Data Availability

This information can be accessed from the corresponding author upon request.
